# Analytically Confirmed Intentional Overdose of the Antidepressant Vortioxetine

**DOI:** 10.1007/s13181-024-01027-8

**Published:** 2024-08-06

**Authors:** Adam Daneshmend, Allon Gould, Simon Hudson, John Robert Howard Archer, Paul Ivor Dargan, David Michael Wood

**Affiliations:** 1 Clinical Toxicology and General Medicine, Guy’s and St Thomas’ NHS Foundation Trust and King’s Health Partners, London, UK; 2 Clinical Toxicology, Faculty of Life Sciences & Medicine, King’s College London, London, UK; 3 LGC ASSURE, Fordham, Cambridgeshire, UK

**Keywords:** Toxicity, Analysis, Vortioxetine, Overdose

## Abstract

**Introduction:**

Vortioxetine is an antidepressant with a multimodal mechanism of action. It is used as a treatment option for patients with major depressive episodes. There have only been two previously reported non-fatal overdoses of vortioxetine; neither of these were analytically confirmed There has also been one case of serotonin syndrome potentially related to vortioxetine and two deaths where vortioxetine was detected. We report here a non-fatal analytically confirmed case of vortioxetine overdose.

**Case report:**

A 32-year-old male presented to the emergency department (ED) 12–13 h after oral ingestion of 1,260 mg of vortioxetine and 350 mg of diazepam. A family member reported that he had been drowsy after the overdose, but his level of consciousness and observations (heart rate, blood pressure and temperature) were normal on review by the pre-hospital emergency services and on arrival to the ED. During a period of observation, he did not develop any features of serotonin syndrome or any other significant toxicity. Toxicological analysis of a blood sample taken in the ED detected vortioxetine (plasma concentration 457 ng/mL 10 h after ingestion) and sub-therapeutic concentrations of diazepam and pregabalin.

**Discussion:**

Despite having a plasma vortioxetine concentration nearly 15-times therapeutic vortioxetine concentrations, this patient did not develop any significant toxicity. In particular he did not develop any clinical or biochemical features of serotonin toxicity that would be expected with this class of antidepressant. Additional reporting of analytically confirmed vortioxetine overdoses will allow clinicians and licensing authorities to further understand the safety of this medication in overdose.

## Introduction

Poisoning and death from antidepressant overdose remains a significant public health issue [1, 2]. This potentially relates to the frequency of their prescription, particularly as they are often prescribed to individuals with a history of deliberate self-poisoning [3]. It is recognised there are differences in the risk of acute toxicity and death not only between different classes of antidepressant drugs, but also within the same antidepressant drug class [4, 5].

Vortioxetine is an antidepressant that has a multimodal mechanism of action distinct from other antidepressants, acting on several serotonin receptor subclasses (antagonist of 5-HT_3_, 5-HT_7_ and 5-HT_1D_ receptors, agonist of the 5-HT_1A_ receptor and partial agonist of the 5-HT_1B_ receptor) and blocking the serotonin transporter (SERT) [6]. The in vivo mechanisms through which vortioxetine enacts its antidepressant, anxiolytic and cognitive enhancing effects are, however, not fully understood. It was licenced for use in the European Union and the USA in 2013 [7, 8]. Since 2015 it has been recommended in the UK as a third-line treatment option for patients with major depressive episodes, at a daily dose of 5 to 20 mg per day [9].

Our understanding of vortioxetine’s potential toxicity is based on limited data, namely: information from the unwanted effects seen in pre-licensing clinical trials, post-marketing surveillance, and five case reports, including two non-fatal non-analytically confirmed overdoses, one case of serotonin syndrome attributed to therapeutic vortioxetine use and two deaths where vortioxetine was detected [10–15]. We present here a case of an analytically confirmed vortioxetine overdose, and in the discussion, we review the previously published information on vortioxetine unwanted effects and toxicity.

## Case Presentation

A 32-year-old white male with a medical history of depression, previous suicide attempts, type-1 diabetes mellitus with associated diabetic retinopathy was found drowsy with multiple packets of empty medication by his mother who called the pre-hospital emergency medical services. On their arrival, 20 min after being called, he was alert with a GCS of 15, blood pressure of 147/95 mmHg, heart rate of 80 beats per minute, respiratory rate of 16, oxygen saturations of 98% on room air and temperature of 37.5 °C. There was a history of ingestion of 63 tablets of vortioxetine 20 mg (total dose 1,260 mg) and 70 tablets of diazepam 5 mg (total dose 350 mg) 12 h previously. His blood sugar was 378 mg/dL (21 mmol/L); he had not taken any of his regular insulin (Lantus and Novorapid) in the last 24 h.

He was transferred to the Emergency Department (ED) and arrived around an hour later; at that time he was still alert and his observations were largely unchanged (blood pressure 156/89 mmHg, heart rate 74 beats per minute, respiratory rate 14 breaths per minute with oxygen saturations of 97% on room air and temperature 37.5 °C), He was evaluated by the medical toxicology team in the ED, where he confirmed the overdose due to worsening of his underlying depression over the previous weeks. He was alert but had a flat affect, with little eye contact and quiet and monotonic speech. Clinical examination was unremarkable; he had normal sized pupils with no ocular clonus, and his peripheral limb tone was normal with normal reflexes and there was no spontaneous or inducible ankle clonus. An electrocardiogram (ECG) showed no abnormalities. Laboratory tests obtained on admission to the ED showed normal renal function, liver function, creatine kinase and complete blood count. His elevated blood sugar was managed with administration of his regular insulin, and his blood sugar normalised prior to discharge. He was observed in the ED and following review by the liaison psychiatry team he was transferred to an inpatient psychiatry bed for ongoing management around 10 h after presentation to the ED. Consent for publication of this case was obtained and provided to the journal in accordance with *JMT* policy.

## Toxicology Analytic Findings

The patient gave informed consent for an additional blood sample to be collected during his period of observation for toxicological analysis. 125 µL of blood was mixed with 1 mL phosphate buffer (1 mol/L, pH 6.3) containing EDDP-D3 (25 µg/L), pregabalin-D4 (100 µg/L), GHB-D6 (500 µg/L). All samples were centrifuged and then prepared for analysis by reversed phase polymeric solid phase extraction using Agilent Nexus sorbent. Prepared samples (10 µL) were analysed on a Thermo XRS ultrahigh-performance liquid chromatography system, interfaced to a Thermo Q Exactive high-resolution accurate mass spectrometer, operating in heated positive ion electrospray mode. Chromatographic separation was achieved in 5.0 min on a Waters Atlantis T3 HPLC column (100 mm x2.1 mm ID, 3 μm) maintained at 40 °C using a gradient elution consisting of a mixture of 0.1% acetic acid and acetonitrile containing 0.1% acetic acid. Data were acquired in full scan mode operating at a mass resolution of 70,000 across a mass range of 50–750 amu. Data dependent scanning was enabled utilising an inclusion list of over 2500 compounds derived from an in-house accurate mass database. A second scan event using all ion fragmentation (AIF) was performed with a stepped higher collisional dissociation (HCD) setting of 15, 35 and 50 at a mass resolution of 35,000 across a scan range of 80–500 amu. Acquired data were processed using Toxfinder software (Thermo) against an in-house database containing over 2500 drugs and metabolites. The presence of reported drugs is confirmed in the data through full scan accurate mass determination of molecular ions, identification of accurate mass qualifier ions in AIF, and the automatic generation of accurate mass tandem mass spectrometry data for comparison with mass spectral libraries.

Vortioxetine was detected and verified by MS2 analysis and the sample re-analysed with a 3 point calibration curve based on the estimated level of vortioxetine from the screen of around 200ng/ml. The concentrations used were 10, 100 and 1000ng/ml. The analysis was performed using the previously described methodology with the levels being calculated using a curve constructed using the response ratios to the EDDP-D3 internal marker derived from the peak areas of the (M + H) + ions of vortioxetine and EDDP-D3. The concentration of vortioxetine detected in the plasma was 457 ng/mL; reported maximum plasma concentration after single and multiple dosing of 20 mg vortioxetine is 8.1 ng/mL and 33.0 ng/mL respectively (the number of multiple doses was not defined by the authors) [16]. Also detected were (i) diazepam (100 ng/mL; therapeutic range 700-1,500 ng/mL) and its metabolites [nordiazepam (20 ng/mL), oxazepam (1 ng/mL), temazepam (6 ng/mL)]; (ii) pregabalin (concentration detected 540 ng/mL; therapeutic range 1000–5000 ng/mL); (iii) benzoylecgonine (5 ng/mL); and (iv) dorzolamide (50 ng/mL).

## Discussion

We have reported here a case of analytically confirmed vortioxetine overdose, with a vortioxetine concentration of 457 ng/mL. The peak concentration (Cmax) of vortioxetine after single and multiple 5, 10 or 20 mg doses of vortioxetine ranged from 1.9 to 8.11 ng/mL and 9 to 33 ng/mL respectively [15, 16]. Although the concentration in our patient was nearly 15-fold that seen in therapeutic use of vortioxetine, there was not any significant toxicity. In particular, he did not develop any evidence of serotonin toxicity that could be expected based on the pharmacological actions of vortioxetine. Although there was reported ingestion of diazepam, the concentration detected on toxicological analysis was sub-therapeutic and so would not have prevented the development of vortioxetine related serotonin toxicity.

There is currently limited information on potential clinical effects associated with overdose of vortioxetine. Information from pre-licensing clinical trials and post-marketing surveillance has reported that the “common unwanted effects” (occurring in between 1% and 10% of individuals) were abnormal dreams, dizziness, gastrointestinal symptoms, pruritis and sweating [6, 15, 17–19]. During pre-licensing clinical trials supra-therapeutic vortioxetine doses of 40 to 75 mg were associated with the common therapeutic unwanted effects, as well as postural dizziness, somnolence and flushing [15]. Post-marketing reports of vortioxetine doses up to 80 mg were associated either no or only mild symptoms [15]. The frequency of more severe unwanted effects reported in post-marketing surveillance (e.g. serotonin syndrome, akathisia and restless leg syndrome) is unknown [15]. One case reported the development of serotonin syndrome in a patient on vortioxetine [10]. A 69-year-old female with a history of psychosis presented with “drenching sweating”, a urinary tract infection and features of serotonin syndrome (hypertension 160/100 mmHg, rigid upper limbs with occasional tremors, brisk reflexes in all limbs, upgoing plantars with limb clonus and an elevated creatine kinase (3,086 units/L)). She was treated with intravenous fluids and clonazepam, but due to worsening agitation, cyproheptadine was administered and she her symptoms resolved five days after the onset of the sweating. It was postulated that the presentation was related to the vortioxetine, although she had been on the same dose (10 mg/day) for over a year. She had also been on amisulpride (800 mg/day); it is unlikely that the amisulpride contributed as it was reported to have been stopped 10 days earlier due to onset of akathisia, but there was no toxicological analysis to exclude the presence of amisulpride.

There have only been two published non-analytically confirmed cases of vortioxetine overdose [11, 12]. A 50-year-old male with major depressive disorder and previous substance use disorder (heroin, cocaine and alcohol) and hepatitis C, took an intentional overdose of 250 mg vortioxetine and 10 mg clonazepam [11]. He presented to the ED around 5 h later and at that time his heart rate, blood pressure, temperature, level of consciousness and neurological examination were normal and there was no evidence of serotonin syndrome. He was observed for 12 h and then discharged to an inpatient psychiatry unit. Urine immunoassay drug testing was positive for “benzodiazepines”; there was no additional toxicological analysis of blood and/or urine undertaken to determine if vortioxetine had been taken. A 20-year-old female with depression ingested a “handful of medications”; her current medicines included vortioxetine, lamotrigine, lurasidone and extended-release bupropion [12]. Of note, her psychiatrist had discontinued escitalopram approximately 2 weeks previously, but some escitalopram tablets were in the room where she was found. On presentation to the ED she had features of serotonin syndrome (pyrexia (38 °C), tachycardia (150 beats per minute), dilated reactive pupils without ocular clonus, hyper-reflexia in her legs and inducible ankle clonus). She was admitted to critical care and treated with diazepam (40 mg/day), midazolam (4–6 mg/hour) and propofol (20–30 mg/hour). Twenty hours after ingestion she developed hypertension (200/110 mmHg) and worsening pyrexia (38–39 °C) with ongoing myoclonus and hyper-reflexia; cyproheptadine was added to her management. Over the next 4 days her symptoms resolved and then she was discharged to an inpatient psychiatry unit. Urine drug testing detected therapeutically used midazolam and diazepam, as well as lamotrigine, escitalopram, and the bupropion metabolite hydroxybupropion; unfortunately, the laboratory was unable to screen for vortioxetine or lurasidone. As it is not possible to confirm vortioxetine ingestion, the detected escitalopram may have been more causative.

In addition, vortioxetine has been detected in two deaths on post-mortem toxicological analysis [13, 14]. A 65-year-old female who had started on vortioxetine (10 mg/day) during a previous mental health admission, was found dead at home [13]. The post-mortem identified undigested tablets in stomach qualitatively confirmed to be vortioxetine and toxicological analysis by LC-MS/MS detected only vortioxetine in post-mortem blood (1,197 ng/mL), brain (804 ng/g), lung (8,992 ng/g), liver (1,389 ng/g) and kidney (292 ng/g) samples. The post-mortem cause of death was recorded as sudden cardiac arrest on a background of chronic ischaemic heart disease; there was no reference to the significance of the detected vortioxetine. A 38-year-old female with underlying depression, treated for 4 months with vortioxetine (10 mg/day) and aripiprazole (15 mg/day), was found to have hanged herself at home [14]. A post-mortem was conducted 3 days after death and toxicological analysis using LC-MS/MS detected aripiprazole in femoral blood (168 ng/mL), along with vortioxetine in femoral blood (234 ng/mL), brain (490 ng/g), lung (479 ng/g), liver (3,571 ng/g), kidney (798 ng/g), bile (2,267 ng/mL) and gastric content (253 ng/g) samples. The authors postulated that the detected concentration of vortioxetine had not caused significant impairment as the individual had committed suicide by hanging.

## Conclusion

Information from pre-licensing clinical trials and post-marketing surveillance suggests that therapeutic and supra-therapeutic dosing of vortioxetine is not associated with severe unwanted effects. There is limited information from non-fatal and fatal cases of vortioxetine overdose to determine the potentially toxic dose of this antidepressant drug. Our patient took a reported overdose of 1,260 mg of vortioxetine, with post-ingestion concentrations nearly 15-fold those seen in regular therapeutic use of vortioxetine. Despite this large analytically confirmed overdose, he did not develop any clinically significant features, and in particular there was no evidence of serotonin syndrome. Additional reporting of analytically confirmed vortioxetine overdoses will allow clinicians and licensing authorities to further understand the safety of this medication in overdose.
